# Evaluation of Lidocaine and Metabolite Pharmacokinetics in Hyaluronic Acid Injection

**DOI:** 10.3390/pharmaceutics13020203

**Published:** 2021-02-02

**Authors:** Ju Hee Kim, Dong Wook Kang, Go-Wun Choi, Sang Bok Lee, Seongjin Lee, Hea-Young Cho

**Affiliations:** 1 College of Pharmacy, CHA University, 335 Pangyo-ro, Bundang-gu, Seongnam-si, Gyeonggi-do 13488, Korea; 20135107@ppharm.org (J.H.K.); dongwk203@gmail.com (D.W.K.); gwchoi153@gmail.com (G.-W.C.); 2 CHA Meditech Co., Ltd., Daejeon-si 1646, Korea; sblee@chamc.co.kr (S.B.L.); sjlee@chamc.co.kr (S.L.)

**Keywords:** lidocaine, monoethylglycylxylidide, glycylxylidide, hyaluronic acid injection, pharmacokinetics, parent-metabolite pharmacokinetic model, modeling

## Abstract

Lidocaine-incorporated hyaluronic acid injection (LHA) is considered a promising way to increase patient compliance. Various reviews and analyses have been conducted to verify that the addition of lidocaine had no effect on the product quality of hyaluronic acid injections. However, possible pharmacokinetic (PK) alterations of lidocaine and its active metabolites, monoethylglycylxylidide (MEGX) and glycylxylidide (GX), in hyaluronic acid injection have not been studied so far. Thus, the objective of this study was to evaluate lidocaine and its metabolite PK after 0.3% lidocaine solution or LHA injection and to investigate any changes in PK profiles of lidocaine and its active metabolites. To do this, a novel bio-analytical method for simultaneous determination of lidocaine, MEGX, and GX in rat plasma was developed and validated. Then, plasma concentrations of lidocaine and its active metabolites MEGX and GX following subcutaneous (SC) injection of 0.3% lidocaine solution or LHA with 0.3–1% lidocaine in male Sprague-Dawley rats were successfully determined. The obtained data were used to develop a parent-metabolite pharmacokinetic (PK) model for LHA injection. The half-life, dose-normalized C_max_, and AUC_inf_ of lidocaine after SC injection of lidocaine solution and LHA did not show statistically significant difference. The PK characteristics of lidocaine after LHA administration were best captured using a two-compartment model with combined first-order and transit absorption and its clearance described with Michaelis–Menten and first-order elimination kinetics. Two one-compartment models were consecutively added to the parent model for the metabolites. In conclusion, the incorporation of lidocaine in hyaluronic acid filler injection did not alter the chemical’s pharmacokinetic characteristics.

## 1. Introduction

The American Society for Aesthetic Plastic Surgery recently reported that more than 1.6 billion dollars were spent on injectables in 2019. Among non-surgical procedures, hyaluronic acid (HA) injection ranked the second highest, with 749,409 procedures conducted in 2019. Hyaluronic acid filler injection is widely used in the field of dermal cosmetics to eradicate hints of the aging process. It is subcutaneously (SC) injected to augment soft tissue and restore facial volume. It is favored over other fillers due to its safe profile, effectiveness, high durability, and reversibility [1,2].

However, there have been consistent patient complaints about pain and discomfort. To increase patient compliance, various topical anesthetic agents and regional anesthesia have been used to ease injection pain. In line with this, several hyaluronic acid injection products incorporated with 0.1–1% lidocaine have been introduced to enhance patient comfort. Since then, several in vitro studies, clinical studies, systemic reviews, and meta-analysis have been conducted to confirm that the addition of lidocaine can decrease pain without affecting product characteristics, HA content, or physical properties [2,3,4]. Nonetheless, pre-clinical studies evaluating changes in pharmacokinetic profiles of lidocaine contained in hyaluronic acid injection to ensure its safety have not been reported.

**Figure 1:**
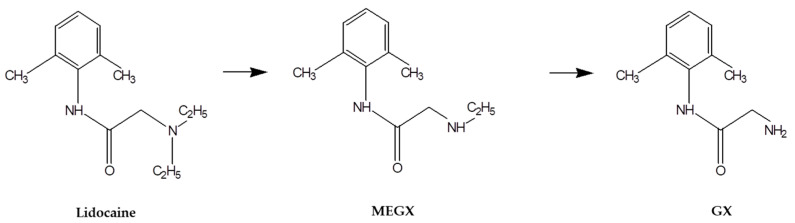
Lidocaine metabolism pathway.

Lidocaine is an amide-group local anesthetic. It is also used as an anti-arrhythmic agent, especially in post-myocardial infarction patients. Lidocaine rapidly and extensively undergoes sequential oxidative N-dealkylation by cytochrome P450 3A4 in the liver to produce two active metabolites, monoethylglycylxylidide (MEGX) and glycylxylidide (GX) [5] (Figure 1). Since its approval, lidocaine has been widely used as human and animal anesthetic or preventive anti-arrhythmic medicine for several decades. Pharmacokinetics of lidocaine following intravenous and extravascular administrations in various species with formulations and dosages have been studied for over 40 years [6]. Although lidocaine has a safe anesthetic profile, further study on pharmacokinetic (PK) alterations of lidocaine is needed to ensure its safety as an adjunct for hyaluronic acid injection.

Additionally, studies have reported that toxic and arrhythmic effects of lidocaine might be partially dependent on its two metabolites MEGX and GX, since they are approximately 80% and 10% as potent anti-arrhythmic agents as lidocaine, respectively [5,7,8]. Although one study has evaluated pharmacokinetics of four compounds including 2,6-xylidine as the third metabolite of lidocaine [9], no other literature has reported its pharmacological activity. Therefore, in the present study, plasma concentrations of lidocaine, MEGX, and GX were determined.

Many studies have reported HPLC, LC, and GC-MS methods for determination of lidocaine [9,10,11,12,13,14,15,16,17,18,19,20,21,22,23,24]. However, only few of them have described bioanalysis method for its metabolites. Saluti et al. [24] have reported on the analysis of lidocaine and one of its metabolites, MEGX, in biological fluids pre-treated with solid-phase extraction method using LC-MS/MS. Bursi et al. [9] have mentioned bioanalysis of lidocaine, MEGX, GX, and 2,6-xylidine with a validated LC-MS/MS method without reporting the details of the method or conditions of the analysis. Maes et al. [23] have previously determined lidocaine, MEGX, and GX concentrations in dog or horse plasma samples using HPLC. However, the method required 1000 µL of plasma and reported rather high limits of quantitation for MEGX and GX, which were 20 and 200 ng/mL, respectively.

To determine concentrations of lidocaine, MEGX, and GX in rat plasma samples, a bioanalysis method using smaller amount of plasma (50 µL) and with lower limit of quantitation was necessary. Thus, a novel bioanalysis method for simultaneous determination of lidocaine, MEGX, and GX was developed and validated for PK evaluation. Then, with obtained data, a PK model for lidocaine and its metabolites following lidocaine-incorporated hyaluronic acid injection (LHA) administration was developed.

HyaFilia Classic PLUS 1.0 is a hyaluronic acid dermal filler injection. It uses hyaluronic acid from *Streptococcus equi* with 1,4-butanediol diglycidyl ether as a cross-linking agent. The objective of this study was to characterize the pharmacokinetics (PK) of lidocaine and its two active metabolites after administration of HyaFilia Classic PLUS 1.0 pre-incorporated with various percentages of lidocaine and to determine any alterations in their behavior.

## 2. Materials and Methods

### 2.1. Chemicals and Reagents

Lidocaine solution (0.3%) and hyaluronic acid filler containing different doses of lidocaine (HyaFilia Classic PLUS incorporated with 0.3, 1, and 3% lidocaine) were obtained from CHA Meditech CO., Ltd. (Yuseong-gu, Korea). Papaverine (internal standard, ISTD) and formic acid were purchased from Sigma Aldrich (St. Louis, MO, USA). Acentonitrile was purchased from J.T. Baker (Phillipsburg, NJ, USA). Distilled water was produced by Evoqua Water Technologies (Pittsburgh, PA, USA). Heparin was purchased from Huons (Gyeonggi-do, Korea).

### 2.2. Animals

Twenty-five male Sprague-Dawley rats (8–12 weeks) were obtained from Orient Bio Inc. (Gyeonggi-do, Republic of Korea). Weights of all animals before drug administration ranged from 342 to 370 g. Two rats were placed in one cage. All rats were housed in a ventilated room with a controlled temperature of 23 ± 2 °C, a relative humidity of 50 ± 10%, and 12 h light/dark cycle. Rats were used for experiments after one week of acclimation. The animal experiment for this study was carried out in the CHA laboratory animal research center of CHA University after obtaining approval from the Institutional Animal Care and Use Committee (IACUC) (IACUC190041, 30 January 2019).

### 2.3. Animal Study Design

The animal study was carried out for PK evaluation of lidocaine and its metabolites in rat plasma samples. Twenty-five rats were randomly allocated into five groups. Two groups of rats were given approximately 0.5 mL of 0.3% lidocaine solution intravenously (Group 1) or subcutaneously (Group 2) to administer 4 mg/kg lidocaine. Three groups of rats were given SC injection of 1 mL LHA with 0.3% lidocaine (Group 3), 1% lidocaine (Group 4), or 3% lidocaine (Group 5), delivering 3, 10, and 30 mg of lidocaine, respectively. Nominal sampling time was determined based on the previously reported half-life of lidocaine (1.2 ± 0.3 h) after IV administration in rats [25]. Considering the possible delayed release effect caused by hyaluronic acid, the final sampling time was set at 12 h. Blood samples were collected via jugular veins into heparinized tubes before administration and at 0.17, 0.33, 0.50, 0.75, 1, 2, 4, 8, and 12 h after administration. Blood samples were centrifuged at 13,000 rpm for 10 min immediately after collection. Plasma was then taken from blood sample and stored at −80 °C. Heparin was used as an anticoagulant.

### 2.4. Analytical Methodology

A novel analysis method was developed for simultaneous determination of lidocaine and two metabolites in rat plasma samples using an Agilent 1290 Infinity II UPLC system coupled with an Agilent 6490 tandem mass spectrometer (Agilent Technologies, Santa Clara, CA, USA). Standard stock solutions of lidocaine, MEGX, GX, and ISTD were individually prepared in 100% methanol at a concentration of 1 mg/mL and stored in a refrigerator (−20 °C). The ISTD solution was prepared in the same manner to a final concentration of 100 ng/mL. A total of 1 mL mixed working solutions of four compounds (lidocaine, MEGX, GX, and ISTD) were prepared by diluting the 100 µL of each compounds’ standard stock solution with 500 µL 50% aqueous methanol. Samples for calibration curves and QC (quality control) were prepared by spiking 5 µL of the standard working solution in 45 µL of blank rat plasma. The final concentrations of the standard calibration curves for all four components ranged between 1 to 500 ng/mL (1, 5, 10, 50, 100, 500 ng/mL). QC samples were prepared at 1 (lower limit of quantitation, LLOQ), 3 (low QC, QL), 80 (mid QC, QM), and 400 (high QC, QH) ng/mL for accuracy and precision evaluation.

Analytes were extracted from rat plasma samples by liquid–liquid extraction with ethyl acetate (EA). First, 50 µL of rat plasma was mixed with 10 µL ISTD solution (100 ng/mL). Next, 600 µL of EA was added to the mixture and vortexed for 3 min using a vortex mixer and centrifuged at 13,000 rpm for 5 min at room temperature. Then, 500 µL of the supernatant was moved to a clean tube and evaporated under nitrogen at 40 °C. After that, the extract was reconstituted with 50 µL of 50% methanol and vortexed for a minute before centrifugation at 13,000 rpm for 5 min. Finally, 5 µL of the supernatant was injected into the ultra-performance liquid chromatograph-MS/MS system.

A Phenomenex Kinetex C18 (2.1 mm, I.D (internal diameter) × 100 mm, 1.7 µm) column was used for chromatographic separation. The mobile phase consisted of 0.1% formic acid in distilled water (solvent A) and 0.1% formic acid in acetonitrile (solvent B). The gradient elution program was as follows—0.0–2.0 min, 10% B; 2.0–2.5 min, 95% B; 2.5–4.0 min, 95% B; 4.0–4.1 min, 10% B; and 4.1–5.0 min, 10% B. The flow rate was set at 0.2 mL/min. The injection volume was 5 µL and the acquisition time was 5 min. The temperature of column was set at 40 ± 5 °C and the auto-sampler sample tray temperature was set at 10 ± 5 °C.

The mass spectrometer was operated in positive electrospray ionization (ESI) mode. Mass transitions for multiple-reaction monitoring (MRM) were—*m*/*z* 235.18 → 86.1 for lidocaine, *m*/*z* 207.15 → 58 for MEGX, *m*/*z* 179.12 → 122 for GX, and *m*/*z* 340.16 → 324.1 for papaverine with collision energies of 17, 13, 13, 29 eV, respectively. Gas temperature, gas flow, nebulizer, and capillary voltage were set at 200 °C, 14 L/min, 20 psi, and 3000 V, respectively. Mass Hunter software (version B.07.01, Agilent Technologies, Santa Clara, CA, USA) was used for data processing after data acquisition.

The developed method for detection of lidocaine and its metabolites in rat plasma was validated in accordance with the Guidance for Industry—Bioanalytical Method Validation by the US Food and Drug Administration (2018).

### 2.5. Pharmacokinetics and Modeling

#### 2.5.1. PK Analysis and Model Development

A PK analysis was performed using Winnonlin software (version 8.1, Certara^TM^ Company, NJ, USA). Then, a PK model was developed for lidocaine and its metabolites following LHA injection. The model was implemented in the Phoenix model in Winnonin with an NLME engine and estimated using the First Order Conditional Estimation-Extended Least Squares (FOCE-ELS).

One- and two-compartment models with first-order, zero-order, absorption, and lag time were tested to describe the disposition of lidocaine and its metabolites after LHA injection. In addition, a combined transit model with first-order absorption was evaluated to better describe absorption phase. In the transit model, the optimal number of transit compartments (*NTR*) and the mean transit time (MTT) were estimated. The transit model introduced three parameters—*K_tr_*, *NTR*, and MTT. Two of these three parameters were estimated. The relationship of these parameters is shown in the following equation:

$$\mathrm{MTT}=\;\frac{\left(NTR+1\right)}{Ktr}$$

The inter-individual variability (IIV) was assessed using an exponential variability model as following equation:

$$P_{i}=P_{\mathrm{tv}}\;\times \exp\;\left(ETA\right)$$

where P_i_ represents the value of the individual PK parameter, P_tv_ is a typical value for P, and *ETA* indicates an interindividual random effect with the mean of zero and variance of ω^2^. Residual random variability (ε) was calculated using a proportional error model for the three types of observations.

Optimal model selection was guided by changes in diagnostic values including twice the negative log like (−2LL), Akaike information Criterion (AIC), Baysian Information Criteria (BIC), visual inspection of various diagnostic plots (goodness of fit plot), and precision of parameter estimates. The visual predictive check (VPC) and boot strap analysis was conducted for non-parametric evaluation of the model.

The final model without absorption was employed when predicting plasma concentration after intravenous injection of 0.3% lidocaine solution.

#### 2.5.2. Model Evaluation

Graphical diagnostics including basic goodness-of-fit plot and other accessory plots were used for single run-based diagnostics during model development. For the final model, the robustness and predictive performance were evaluated using multiple run-based diagnostics such as bootstrapping. A bootstrap procedure was conducted with a total of 1000 bootstrap-resampled datasets from the original dataset. Bootstrap results of median and 95% confidence intervals (CIs, 5th and 95th percentiles) were compared with final parameter estimates. The 95% confidence intervals of parameters obtained from this step were compared with final parameter estimates.

### 2.6. Statistical Analysis

Statistical significance was evaluated through Wilcoxon rank summation test with R software (R Foundation for Statistical Computing, Vienna, Austria) with *p* < 0.05 inferring significant difference.

## 3. Results

### 3.1. Analytical Method Development and Validation

**Figure 2:**
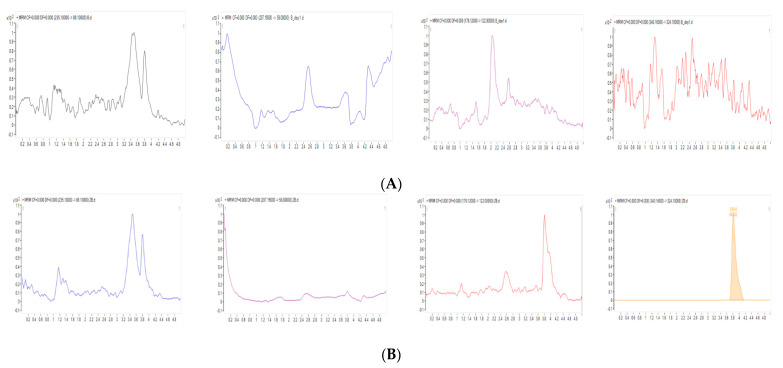

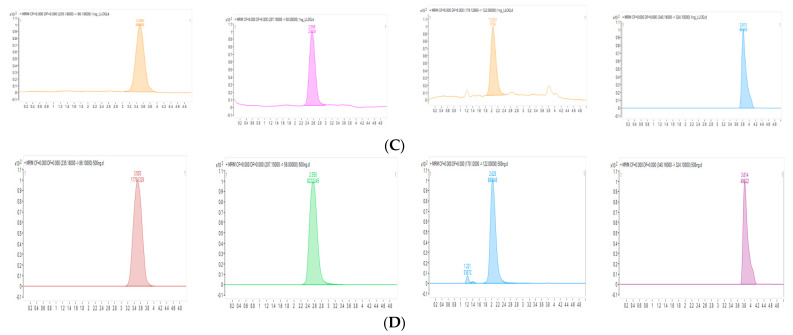
Representative chromatograms of Blank (**A**), Zero-blank (**B**), Lower limit of quantitation (LLOQ) (**C**), and Upper limit of quantitation (ULOQ) (**D**) calibration standard samples. The x-axis and y-axis represent the retention time (min) and relative response, respectively.

Representative MRM chromatograms of zero-blank plasma, LLOQ, and ULOQ are shown in Figure 2. There were no significant interferences from endogenous substances around retention times of analytes in blank plasma, indicating sufficient specificity of the method. Retention times of lidocaine, MEGX, GX, and the ISTD were 3.5, 2.6, 2.0, and 3.8 min, respectively.

Linearity for lidocaine, MEGX, and GX in rat plasma was excellent over a concentration range of 1–500 ng/mL. Calibration curves fitted well with correlation coefficients (r^2^) exceeding 0.99. Typical linear regression equations were as follows—y = (5.248 ± 0.631) x + (0.079 ± 0.085) for lidocaine, y = (2.723 ± 0.288) x + (0.074 ± 0.033) for MEGX, and y = (0.214 ± 0.015) x + (0.003 ± 0.004) for GX. Table 1 summarizes precision and accuracy data for lidocaine, MEGX, and GX at 1 (LLOQ), 3 (QL), 80 (QM), and 400 (QH) ng/mL in rat plasma. Intra-batch accuracy ranged from 91.85% to 100.99% for lidocaine, 92.73% to 104.14% for MEGX, and 98.38% to 130.62% for GX. The coefficient of variation (CV) was less than 13.94% for lidocaine, 12.17% for MEGX, and 11.07% for GX. The inter-batch accuracy ranged from 92.67% to 107.35% for lidocaine, 89.21% to 103.69% for MEGX, and 99.91% to 108.76% for GX. The coefficient of variation was within 11.68% for lidocaine, 9.14% for MEGX, and 8.03% for GX. All values were within acceptable criteria. Therefore, this method showed suitable precision, accuracy, and reproducibility in compliance with FDA guidelines.

This is the first report on a simultaneous bioanalysis method of lidocaine, MEGX, GX that used only 50 µL of matrix and with all compounds’ LLOQ as low as 1 ng/mL. This simultaneous bioanalysis method of lidocaine and its two active metabolites in LC-MS/MS was developed after several trials. First, various columns including Intersil ODS-3 (2.1 × 100 mm, 5 µm), Hypersil GOLD (2.1 × 100 mm, 1.9 µM), and Phenomenex Kinetex C18 (2.1 × 100 mm, 1.7 µm) were tried to obtain the most appropriate chromatograms for four compounds (lidocaine, MEGX, GX, and ISTD). When Intersil ODS-3 column was used, GX was not detected. With the Hypersil GOLD coloumn, GX was detected. However, there was an unknown residue at the retention time of GX. Therefore, Phenomenex Kinetex C18 was used, since all analytes including GX were detected with appropriate sensitivity and selectivity, which can be confirmed in the third column of Figure 2. Second, the pre-treatment method was tested. Between protein precipitation and liquid–liquid extraction (LLE), LLE showed higher sensitivity. Next, different solvents (ethyl acetate, methylene chloride, and methyl tert-butyl ether) were tested for LLE. Among these tested solvents, ethyl acetate was chosen since it resulted in clearer blank with sharper and more symmetric chromatograms for all analytes as presented in Figure 2. Last, several gradient conditions were tested to optimize retention time of each analyte. The final gradient condition produced symmetric peak shapes for all analytes and sufficiently separated all four compounds (lidocaine, MEGX, GX, and ISTD) within a short retention time of 5 min.

### 3.2. PK Model Development and Model Evaluation

Non-compartmental analysis of data was conducted to acquire initial parameters with which compartment modeling was sequentially performed. Elimination phase of lidocaine after SC administration of LHA showed a bi-exponential decay, indicating a two-compartment disposition model.

A two-step method was used to build the optimal model for the observed data of LHA. First, several models were fitted to plasma concentration-time data of lidocaine. One- and two-compartment models with various combinations of zero-, first-order, and transit absorption models were tested to describe lidocaine profile following LHA administration. Clearance was tested as linear and non-linear Michaelis–Menten type. After that, two one-compartment models for metabolites MEGX and GX were added to complete the parent-metabolite pharmacokinetic model.

**Figure 3:**
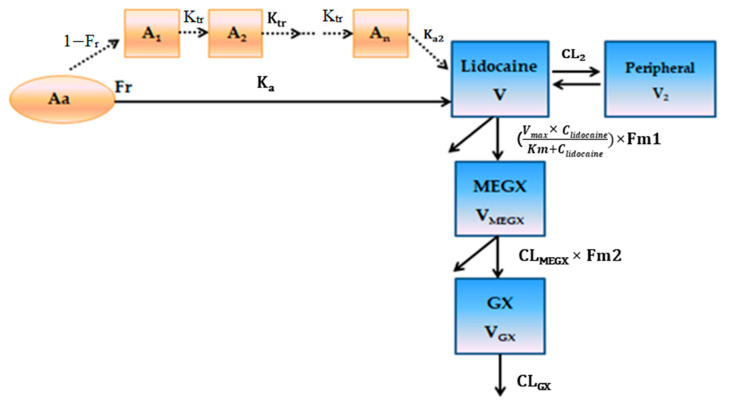
Schematic PK model for lidocaine and its metabolites following the subcutaneous (SC) administration of LHA.

The optimal model was selected according to the method described in the method Section 2.5.1. A brief summary of diagnostic values of −2LL, AIC, and BIC of several models tried are presented in Table 2. The two-compartment model that combined 1st order and transit absorption with non-linear clearance (M202) was identified as the optimal model to describe plasma profiles of lidocaine in rats after SC administration of LHA injection. The final PK model of lidocaine and the two metabolites is depicted in Figure 3.

Primary parameters such as half-life, maximum plasma concentration (C_max_), time of C_max_ (T_max_), and area under the plasma concentration-time curve (AUC_inf_) of lidocaine, MEGX, and GX for each group are summarized in Table 3. Model-estimated parameters with bootstrap results are presented at the Table 4. All estimated values were well within 25–95% CI bootstrap values with reasonable CV (%).

**Figure 4:**
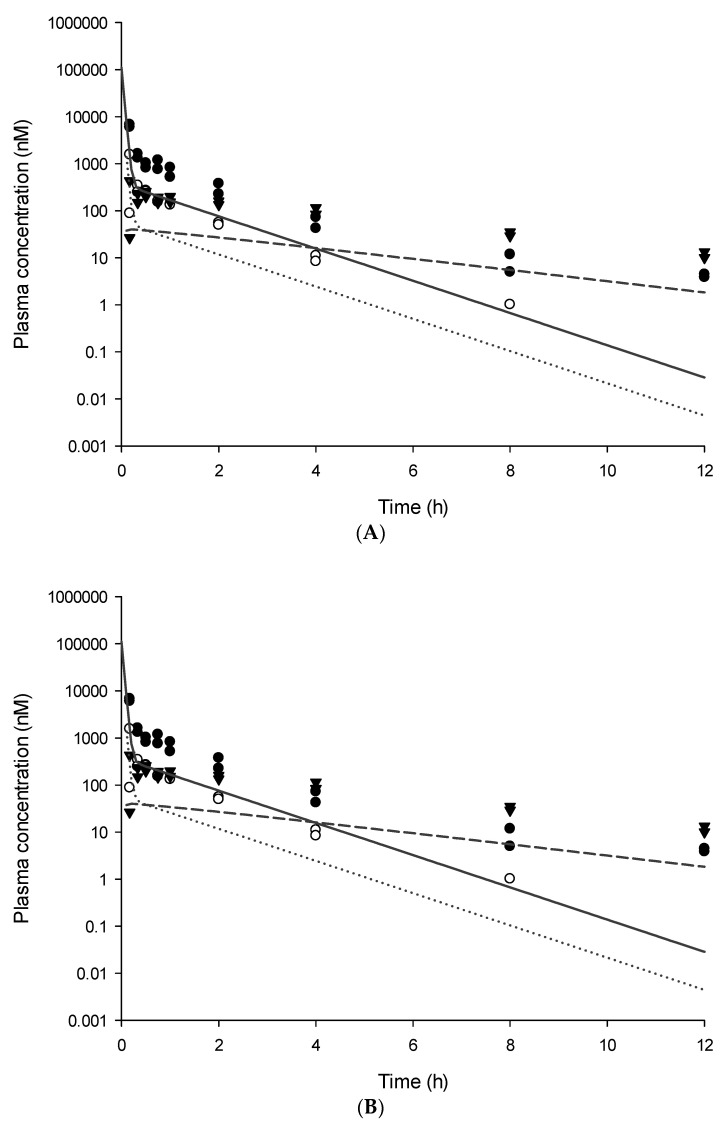

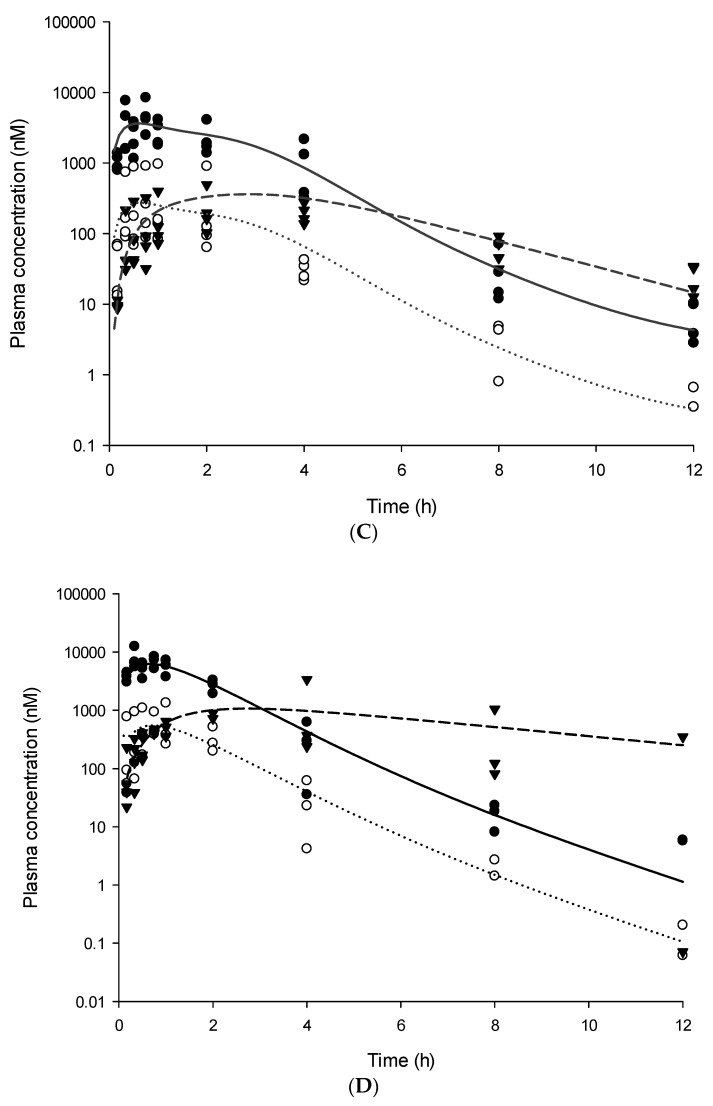

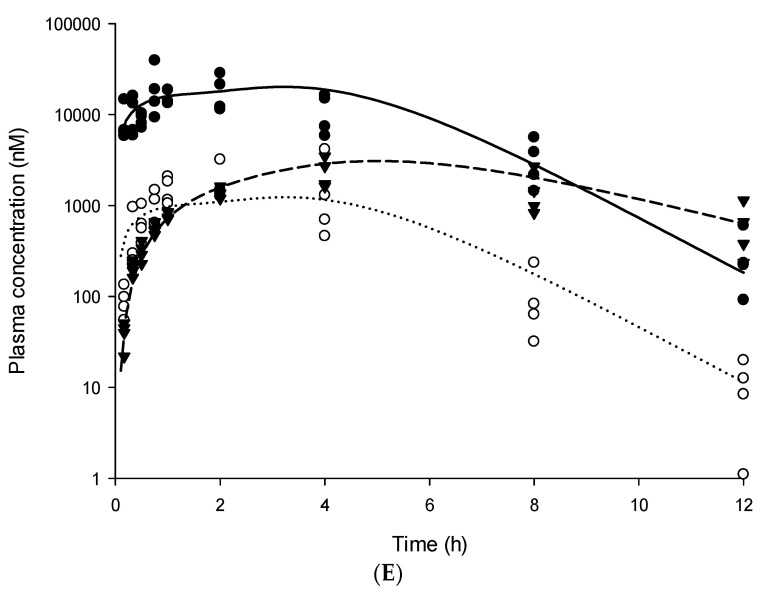
Predicted plasma concentration-time plot of Lidocaine (solid line, ●), MEGX (dotted line, ▼), and GX (dashed line, ○) with observed data after SC administration of 0.3% lidocaine solution IV (**A**), 0.3% lidocaine solution SC (**B**), 0.3% LHA (**C**), 1% LHA (**D**), or 3% LHA (**E**).

The model-predicted plasma concentration-time plots fitted to the observed data of each group are shown in Figure 4.

**Figure 5:**
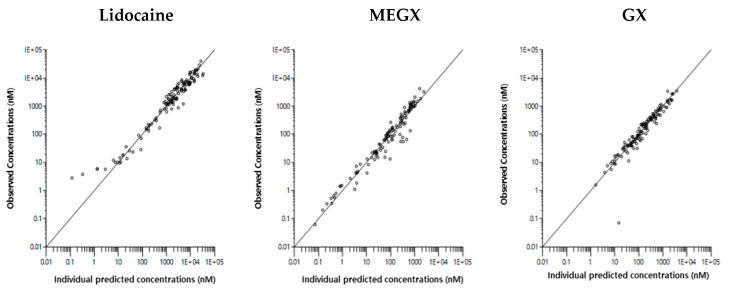

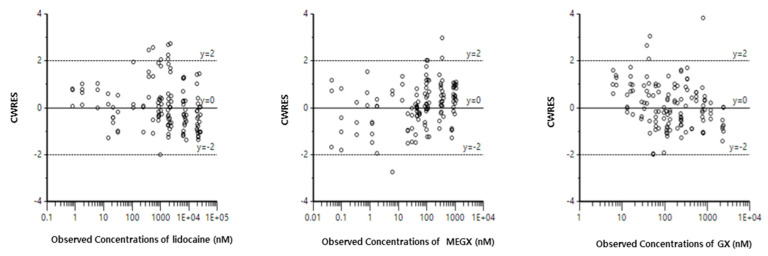
Goodness-of-fit plots for the final model. Individual predicted concentrations of lidocaine, MEGX, and GX vs. observations (upper) and conditional weighted residuals (CWRES) vs. observations (lower). Circles represent the observed lidocaine, MEGX, and GX concentrations.

**Figure 6:**
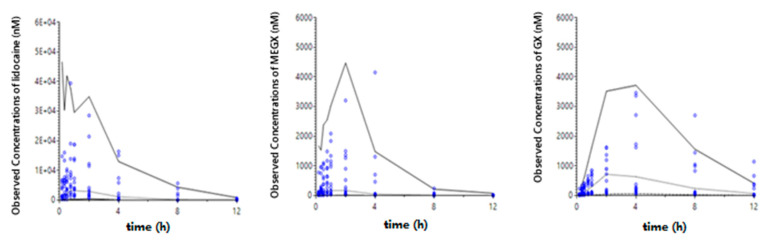
Visual predictive check plot of the final model. A total of 1000 datasets were simulated using the final PK parameter estimates. Circles represent the observed lidocaine, MEGX, and GX concentrations. The lines in each graph represent 5th, median, and 95th percentiles of the simulated concentrations.

The final model was evaluated by means of goodness-of-fit plots and visual predictive check. As seen in Figure 5, no significant bias can be detected in the model and the model was able to capture the general trend of the data adequately. Figure 6 presents the visual predictive check plots for the final model. In addition, the robustness and predictive performance of the final model were evaluated using bootstrapping. Bootstrap results of lower and upper 95% confidence intervals (CIs) are provided in Table 3. All final parameter estimates were within the 95% confidence interval.

## 4. Discussion

This study aimed to examine the possibilities of lidocaine interacting with hyaluronic acid. Although commonly used for almost decades, several papers were published to raise awareness on lidocaine local anesthetic toxicity. There has been a report on a female patient who experienced bradycardia followed by pulseless electrical activity shortly after being given 60 mL of 2% lidocaine with 1% epinephrine [26]. Also, systemic toxicity including cardiotoxic and neurotoxic adverse events was reported after application of topical anesthetics, 30% lidocaine gel, which is considered relatively safe [27]. Therefore, although small in volume, investigating alterations in lidocaine pharmacokinetics when injected with hyaluronic acid is crucial.

The Sprague–Dawley rat was chosen for the experiment since not only is the species considered a primary rodent species for toxicity testing by EMA, but also the species is often used in pharmacokinetic experiments. In addition to this, there were several literatures reporting that humans and rats are considered to share similar lidocaine metabolic pathways. Imaoka et al. [28] have reported that in both human and man, the majority of the lidocaine dose is metabolized with cytochrome P450. Also, Leclercq et al. [29] have stated that rats and humans share great similarities in lidocaine metabolism.

According to the good practice guide [30], the ideal volume of SC and intravenous (bolus) administration in rat is 5 mL/kg. However, in Rat and Mouse Anesthesia and Analgesia—Formulary and General Drug Information [31], lidocaine solution was recommended to be diluted under 0.5% before injection and to not exceed 7 mg/kg total dose, SC or intra-incisional. Thus, to avoid toxicity during pharmacokinetic experiments, the administration volume was adjusted for intravenous and SC injection of 0.3% lidocaine solution, so that the administered dose would equal only 4 mg/kg. For the LHA administration group, the whole product of Hyafilia Classic PLUS 1.0 (1 mL) injection was administered. Therefore, in the case of 0.3% LHA, about 8 mg/kg (8.10-8.60 mg/kg depending on rats’ weights) was administered.

In order to directly compare the the C_max_ and AUC_inf_ of the Group 2 and 3, dose-normalization had to be conducted in order to directly compare the the C_max_ and AUC_inf_ of Group 2 and 3 since different doses were administered. The dose-normalized C_max_ and AUC_inf_ was calculated in Winnonlin software (version 8.1, Certara^TM^ Company, NJ, USA) by dividing lidocaine C_max_ and AUC_inf_ values by the administered dose of lidocaine. The mean and the standard deviation of the dose-normalized C_max_ (C_o_) and AUC_inf_ of lidocaine for each group were as follows—1677.66 ± 522.35 ng/mL and 307.19 ± 21.80 ng×h/mL for Group 1; 135.62 ± 25.55 ng/mL and 214.01 ± 20.67 ng×h/mL for Group 2; 144.31 ± 66.55 ng/mL and 310.69 ± 108.57 ng×h/mL for Group 3; 71.93 ± 19.72 ng/mL and 111.20 ± 12.03 ng×h/mL for Group 4; 76.64 ± 27.45 ng/mL and 271.42 ± 68.15 ng×h/mL for Group 5, respectively. The dose-normalized lidocaine C_max_ and AUC_inf_ for Groups 2 and 3 did not show statistically significant difference; therefore, it could be said that lidocaine incorporation in hyaluronic acid did not alter its pharmacokinetics characteristics. However, to understand the PK characteristics of lidocaine and its active metabolites in LHA further, parent-metabolite modeling was conducted.

Several models (Table 2) were then tested to find the best fitted model for the observed data. The plasma concentration of lidocaine was best described by the two-compartment model with combined first and transit model absorption and with Michaelis–Menten clearance. Its metabolites were described with a simple one-compartment model. Lidocaine pharmacokinetics was often described with two-compartment models in previous reports [20,32,33], which is in agreement with the final model. In the *xy*-plot of plasma concentration-time data for SC LHA administration group, multiple peaks were observed in the absorption phase absorption of lidocaine. Therefore, combined first-order and transit model absorption were implemented as described in previous reports [34] to better fit the observed values.

The MM equation was employed in explaining the elimination of lidocaine after SC injection of LHA for the following three reasons—(1) the areas under the concentration-time curves verses dose plots of lidocaine in LHA showed exponential increase; (2) lidocaine is known to be metabolized into MEGX via enzymes in vivo; and (3) the lower model diagnostic values (−2LL, AIC, and BIC) yielded for model M202. Furthermore, there were in vitro reports such as Deshpande et al. that have described the microsomal metabolism of lidocaine to MEGX using the MM equation [35]. The in vitro Km values for the metabolism of lidocaine to MEGX by rat liver microsome and rat olfactory microsomes were reported as 304.33 and 156.77 µM, respectively.

In the developed model, the estimated Km value in vivo for the metabolism of lidocaine to MEGX was 136.81 µM. Linear pharmacokinetic behavior is manifested when the plasma concentration of a drug becomes smaller than 0.1 km [36]. Thus, the lidocaine clearance after the SC LHA administration was better described using MM clearance along with first-order elimination, since the plasma concentrations of lidocaine far exceeds 13.68 µM (10% of the estimated Km values) around the absorption phase after the administration of LHA with 3% lidocaine. Since the T_max_ of lidocaine and MEGX are very close (please refer to Table 3), it could be understood that the metabolism of lidocaine into MEGX is extremely rapid and mostly happens within the first few hours of administration. Therefore, after LHA injection with 3% lidocaine, the metabolism of lidocaine to MEGX can be explained using the MM equation; thus, manifesting non-linear elimination pattern of lidocaine after the SC injection with the range of 0.3–3% lidocaine.

The fraction of conversion (Fm1, Fm2) was multiplied to the cleared amount of each compound, and it was predicted that about 64% of lidocaine was converted into MEGX and about 47% MEGX was converted into GX when hyaluronic acid pre-incorporated lidocaine was injected subcutaneously.

## 5. Conclusions

In this study, data collected from rats after SC administration of lidocaine solution or LHA were used to build a pharmacokinetic model to predict concentrations of lidocaine and its two metabolites simultaneously. Using plasma concentration-time data obtained through a novel simultaneous bioanalysis method of these three compounds, a model providing reliable estimates of PK parameters was developed. The model was validated using goodness-of-fit plots. The incorporation of lidocaine into hyaluronic acid filler for injection manifested no statistically significant PK alterations of the compound in rats.

## Figures and Tables

**Table 1:** Accuracy and precision for detection of lidocaine, monoethylglycylxylidide (MEGX), and glycylxylidide (GX).

Lidocaine			
Theoretical Conc. (ng/mL)	Measured Conc. (Mean ± S.D., ng/mL)	Precision (CV, %)	Accuracy (%)
Inter-day			
1	1.07 ± 0.07	6.90	107.35
3	3.17 ± 0.14	4.27	105.78
80	79.81 ± 9.33	11.68	99.77
400	370.68 ± 7.55	2.04	92.67
Intra-day			
1	0.99 ± 0.14	13.94	98.80
3	3.03 ± 0.15	5.09	100.99
80	77.47 ± 3.19	4.11	96.83
400	367.38 ± 18.42	5.01	91.85
MEGX			
Theoretical Conc. (ng/mL)	Measured Concentration (Mean ± S.D., ng/mL)	Precision (CV, %)	Accuracy (%)
Inter-day			
1	0.97 ± 0.09	9.14	96.70
3	3.11 ± 0.17	5.47	103.61
80	82.95 ± 4.82	5.81	103.69
400	356.84 ± 8.01	3.98	89.21
Intra-day			
1	1.00 ± 0.10	9.72	99.50
3	3.12 ± 0.17	5.55	104.14
80	77.87 ± 9.48	12.17	97.34
400	370.94 ± 33.10	8.92	92.73
GX			
Theoretical Conc. (ng/mL)	Measured Conc. (Mean ± S.D., ng/mL)	Precision (CV, %)	Accuracy (%)
Inter-day			
1	1.09 ± 0.05	4.70	108.76
3	3.07 ± 0.25	8.03	102.35
80	79.93 ± 5.58	6.98	99.91
400	402.79 ± 16.76	4.16	100.70
Intra-day			
1	1.03 ± 0.09	8.21	103.62
3	3.00 ± 0.11	3.82	100.02
80	78.70 ± 8.71	11.07	98.38
400	421.79 ± 37.28	8.84	105.45

**Table 2:** Pharmacokinetics (PK) model development process for lidocaine-incorporated hyaluronic acid injection (LHA) administration groups.

Model	Model Description	−2LL	AIC	BIC
100	One compartment model with zero-order absorption	2055.87	2071.87	2094.63
101	One compartment model with first-order absorption	2030.97	2046.97	2069.66
102	One compartment model with first-order absorption with lag time	2042.75	2062.75	2091.11
103	One compartment model with first-order and zero-order absorption	2049.48	2073.48	2102.65
104	One compartment model with transit model absorption	2042.76	2066.76	2100.79
105	One compartment model with zero-order and transit model absorption	2032.19	2064.19	2109.57
106	One compartment model with combined first-order and transit model absorption	2026.92	2058.92	2104.30
200	Two compartment model with first-order absorption	2028.62	2052.62	2081.84
201	Two compartment model combined first-order and transit model absorption	2019.79	2059.79	2121.83
202	Two compartment model combined first-order and transit model absorption with non-linear clearance using Michaelis-Menten equation	2015.44	2059.44	2116.52

**Table 3:** Pharmacokinetic parameters of lidocaine, MEGX and GX (Mean, (S.D.)).

Group	Compound	Half-life (h)	C_max_ or C_o_ (ng/mL)	T_max_ (h)	AUC_inf_ (h × ng/mL)
Group 1	Lidocaine	1.34 (0.06)	6710.62 (2089.38)	-	1228.75 (87.41)
(0.3% solution, IV)	MEGX	0.88 (0.16)	197.49 (177.72)	0.25 (0.11)	97.38 (29.15)
	GX	2.58 (0.03)	61.78 (22.44)	0.33 (0.24)	1786.53 (32.59)
Group 2	Lidocaine	1.44 (0.07)	542.48 (102.20)	0.61 (0.24)	856.03 (82.67)
(0.3% solution, SC)	MEGX	1.60 (0.32)	11.77 (11.08)	0.78 (0.39)	27.23 (16.80)
	GX	3.19 (0.11)	14.71 (4.52)	2.00 (0)	108.14 (47.37)
Group 3	Lidocaine	1.20 (0.13)	1204.21 (535.43)	0.58 (0.20)	2588.77 (847.10)
(LHA 0.3%, SC)	MEGX	0.98 (0.35)	80.03 (80.50)	0.81 (0.24)	197.19 (219.90)
	GX	2.81 (0.89)	44.87 (29.13)	3.00 (1.16)	270.83 (102.72)
Group 4	Lidocaine	0.94 (0.22)	2034.27 (616.60)	0.54 (0.24)	3088.04 (267.43)
(LHA 1%, SC)	MEGX	0.75 (0.35)	132.13 (95.55)	0.75 (0.20)	295.73 (177.43)
	GX	1.64 (0.92)	143.95 (21.66)	2.67 (1.15)	1442.35 (1533.06)
Group 5	Lidocaine	1.52 (0.19)	6332.16 (2168.15)	1.44 (0.66)	22470.67 (5474.96)
(LHA 3%, SC)	MEGX	1.12 (0.22)	469.36 (266.15)	2.25 (1.26)	1979.03 (1477.58)
	GX	4.05 (0.65)	424.31 (155.67)	4.00 (0)	3811.32 (1839.31)

**Table 4:** Estimated PK parameters of lidocaine, MEGX and GX after LHA administration using non-linear mixed-effect modeling.

Parameter (Unit)	Definition	Estimate	CV (%)	Bootstrap 95% CI
				(Lower, Upper)
Fixed effect				
K_a1_ (h^−1^)	Absorption rate constant of first-order absorption	5.92	12.97	(5.19, 6.64)
V_1_/F (L)	Apparent volume of distribution of compartment 1	2.57	13.66	(2.23, 2.89)
V_2_/F (L)	Apparent volume of distribution of compartment 2	0.07	3.22	(0.06, 0.07)
CL_d_/F (L/h)	Inter-compartmental Clearance	0.13	0.37	(0.1323, 0.1332)
Fr	Fraction of the dose absorbed by first-order absorption	0.373	1.19	(0.371, 0.374)
MTT (h)	Mean Transit Time	0.64	11.35	(0.57, 0.70)
Ntr	Number of transit compartments	4.97	0.17	(4.96, 4.98)
Ka_2_(h^−1^)	Absorption rate constant from the final transit compartment to the central compartment	1.67	4.73	(1.59, 1.74)
V_max_ (nmol/h)	Maximum rate of reaction	423,962.94	10.05	(383,479.15, 464,446.73)
Km (nmol/L)	Michaelis-Menten constant	136,808.67	18.62	(112,613.9, 161,003.44)
CL_m1_/F (L/h)	Apparent Metabolite (MEGX) clearance	14.94	15.31	(12.76, 17.11)
Fm1	Fraction of the parent converted to first metabolite	0.65	1.06	(0.644, 0.646)
CL_m2_/F (L/h)	Apparent Metabolite clearance	1.09	4.58	(1.04, 1.14)
Fm2	Fraction of the first metabolite converted to second metabolite	0.47	13.67	(0.46, 0.47)
Random effects				
ω _V_	IIV of V	0.13	9.23	(0.127, 0.152)
ω _CLd_	IIV of CLd	0.19	6.84	(0.181, 0.208)
ω _Vmax_	IIV of Vmax	0.08	7.50	(0.077, 0.089)
ω _Km_	IIV of Km	0.17	7.06	(0.159, 0.183)
ω _CLm1_	IIV of CLm	0.38	6.84	(0.350, 0.402)
ω _CLm2_	IIV of CLm2	0.23	6.96	(0.216, 0.248)
Residual error				
ε_1_	Proportional error of Lidocaine	0.49	2.02	(0.485, 0.504)
ε_2_	Proportional error of MEGX	0.58	3.20	(0.562, 0.597)
ε_3_	Proportional error of GX	0.41	3.99	(0.399, 0.430)

## Data Availability

Not applicable.

## References

[1] Wang C., Luan S., Panayi A.C., Xin M., Mi B., Luan J. (2018). Effectiveness and Safety of Hyaluronic Acid Gel with Lidocaine for the Treatment of Nasolabial Folds: A Systematic Review and Meta-analysis. Aesthetic Plast. Surg..

[2] Levy P.M., De Boulle K., Raspaldo H. (2009). Comparison of Injection Comfort of a New Category of Cohesive Hyaluronic Acid Filler with Preincorporated Lidocaine and a Hyaluronic Acid Filler Alone. Dermatol. Surg..

[3] Raspaldo H., De Boulle K., Levy P.M. (2010). Longevity of effects of hyaluronic acid plus lidocaine facial filler. J. Cosmet. Dermatol..

[4] Matarasso S.L., Carruthers J.D., Jewell M.L. (2006). Consensus Recommendations for Soft-Tissue Augmentation with Nonanimal Stabilized Hyaluronic Acid (Restylane). Plast. Reconstr. Surg..

[5] Narang P.K., Crouthamel W.G., Carliner N.H., Fisher M.L. (1978). Lidocaine and its active metabolites. Clin. Pharmacol. Ther..

[6] Choquette A., Troncy E., Guillot M., Varin F., Del Castillo J.R.E. (2017). Pharmacokinetics of Lidocaine Hydrochloride Administered with or without Adrenaline for the Paravertebral Brachial Plexus Block in Dogs. PLoS ONE.

[7] Burney R.G., DiFazio C.A., Peach M.J., Petrie K.A., Silvester M.J. (1974). Anti-arrhythmic effects of lidocaine metabolites. Am. Hear. J..

[8] Bennett P.N., Aarons L.J., Bending M.R., Steiner J.A., Rowland M. (1982). Pharmacokinetics of lidocaine and its deethylated metabolite: Dose and time dependency studies in man. J. Pharmacokinet. Biopharm..

[9] Bursi R., Piana C., Grevel J., Huntjens D., Boesl I. (2017). Evaluation of the Population Pharmacokinetic Properties of Lidocaine and its Metabolites After Long-Term Multiple Applications of a Lidocaine Plaster in Post-Herpetic Neuralgia Patients. Eur. J. Drug Metab. Pharmacokinet..

[10] Júnior E.R., Bentley M.V.L.B., Marchetti J.M. (2002). HPLC assay of lidocaine in in vitro dissolution test of the Poloxamer 407 gels. Rev. Bras. Ciências Farm..

[11] Malenović A., Medenica M., Ivanović D., Jancic B., Markovic S. (2005). Development and validation of RP–HPLC method for cetrimonium bromide and lidocaine determination. Farmaco.

[12] Zivanovic L., Zecevic M., Markovic S., Petrovic S., Ivanovic I. (2005). Validation of liquid chromatographic method for analysis of lidocaine hydrochloride, dexamethasone acetate, calcium dobesilate, buthylhydroxyanisol and degradation product hydroquinone in suppositories and ointment. J. Chromatogr. A.

[13] Chik Z., Lee T.D., Holt D.W., Johnston A., Tucker A.T. (2006). Validation of High-Performance Liquid Chromatographic—Mass Spectrometric Method for the Analysis of Lidocaine in Human Plasma. J. Chromatogr. Sci..

[14] Abdelwahab N.S. (2013). Determination of Thiomersal, Lidocaine and Phenylepherine in their Ternary Mixture. J. Chromatogr. Sep. Tech..

[15] Belal T.S., Bedair M.M., Gazy A.A., Guirguis K.M. (2015). Validated Selective HPLC-DAD Method for the Simultaneous Determination of Diclofenac Sodium and Lidocaine Hydrochloride in Presence of Four of Their Related Substances and Potential Impurities. Acta Chromatogr..

[16] Grigoriev A., Nikitina A., Yaroshenko I.S., Sidorova A. (2016). Development of a HPLC–MS/MS method for the simultaneous determination of nifedipine and lidocaine in human plasma. J. Pharm. Biomed. Anal..

[17] Al Nebaihi H.M., Primrose M., Green J.S., Brocks D.R. (2017). A High-Performance Liquid Chromatography Assay Method for the Determination of Lidocaine in Human Serum. Pharmaceutics.

[18] Meshram D.B., Mehta K., Mishra P. (2018). Stability Indicating Analytical Method for the Simultaneous Estimation of Lidocaine and Nifedipine in the Combined Dosage Form. Der Pharma Chem..

[19] Yadlapalli S.S.R., Katari N.K., Surendrababu M.S., Karra V.K., Kommineni V., Jonnalagadda S.B. (2019). Simultaneous quantification of lidocaine and prilocaine in human plasma by LC-MS/MS and its application in a human pharmacokinetic study. Pract. Lab. Med..

[20] Schnider T.W., Gaeta R., Brose W., Minto C.F., Gregg K.M., Shafer S.L. (1996). Derivation and Cross-validation of Pharmacokinetic Parameters for Computer-controlled Infusion of Lidocaine in Pain Therapy. Anesthesiology.

[21] Burm A.G., de Boer A.G., van Kleef J.W., Vermeulen N.P., de Leede L.G., Spierdijk J., Breimer D.D. (1988). Pharmacokinetics of lidocaine and bupivacaine and stable isotope labelled analogues: A study in healthy volunteers. Biopharm. Drug Dispos..

[22] Le Normand Y., De Dieuleveult C., Athouel A., Queinnec M., De Villepoix C., Larousse C. (1989). Pharmacokinetics of Lidocaine and Bupivacaine in Retrobulbar and Facial Block. Fundam. Clin. Pharmacol..

[23] Maes A., Weiland L., Sandersen C., Gasthuys F., De Backer P., Croubels S. (2007). Determination of lidocaine and its two N-desethylated metabolites in dog and horse plasma by high-performance liquid chromatography combined with electrospray ionization tandem mass spectrometry. J. Chromatogr. B Anal. Technol. Biomed. Life Sci..

[24] Saluti G., Giusepponi D., Moretti S., Di Salvo A., Galarini R. (2016). Flexible Method for Analysis of Lidocaine and Its Metabolite in Biological Fluids. J. Chromatogr. Sci..

[25] Liu J., Lv X. (2014). The Pharmacokinetics and Pharmacodynamics of Lidocaine-Loaded Biodegradable Poly(lactic-co-glycolic acid) Microspheres. Int. J. Mol. Sci..

[26] Hasan B., Asif T., Hasan M. (2017). Lidocaine-Induced Systemic Toxicity: A Case Report and Review of Literature. Cureus.

[27] Marra D.E., Yip D., Fincher E.F., Moy R.L. (2006). Systemic Toxicity from Topically Applied Lidocaine in Conjunction with Fractional Photothermolysis. Arch. Dermatol..

[28] Imaoka S., Enomoto K., Oda Y., Asada A., Fujimori M., Shimada T., Fujita S., Guengerich F.P., Funae Y. (1990). Lidocaine metabolism by human cytochrome P-450s purified from hepatic microsomes: Comparison of those with rat hepatic cytochrome P-450s. J. Pharmacol. Exp. Ther..

[29] Leclercq I., Saliez A., Wallemacq P.E., Horsmans Y., Lambotte L. (1997). The monoethylglycinexylidide test does not correctly evaluate lidocaine metabolism after ischemic liver injury in the rat. Hepatology.

[30] Diehl K.-H., Hull R., Morton D., Pfister R., Rabemampianina Y., Smith D., Vidal J.-M., Van De Vorstenbosch C. (2001). A good practice guide to the administration of substances and removal of blood, including routes and volumes. J. Appl. Toxicol..

[31] Adams S., Pacharinsak C., Animal Committee, U. o. B. C. (2015). Rat and Mouse Anesthesia and Analgesia: Formulary and General Drug Information. Curr. Protoc. Mouse Biol..

[32] Hsu Y.-W., Somma J., Newman M.F., Mathew J.P. (2011). Population Pharmacokinetics of Lidocaine Administered During and After Cardiac Surgery. J. Cardiothorac. Vasc. Anesthesia.

[33] Greenblatt D.J. (1976). Pharmacokinetic Approach to the Clinical Use of Lidocaine Intravenously. JAMA J. Am. Med Assoc..

[34] Savic R.M., Jonker D.M., Kerbusch T., Karlsson M.O. (2007). Implementation of a transit compartment model for describing drug absorption in pharmacokinetic studies. J. Pharmacokinet. Pharmacodyn..

[35] Deshpande V.S., Genter M.B., Jung C., Desai P.B. (1999). Characterization of lidocaine metabolism by rat nasal microsomes: Implications for nasal drug delivery. Eur. J. Drug Metab. Pharmacokinet..

[36] Van Ginneken C.A.M., Van Rossum J.M., Fleuren H.L.J.M. (1974). Linear and nonlinear kinetics of drug elimination I. Kinetics on the basis of a single capacity-limited pathway of elimination with or without simultaneous supply-limited elimination. J. Pharmacokinet. Biopharm..

